# In vitro toxicological assessment of gadolinium (III) chloride in V79–4 fibroblasts

**DOI:** 10.1186/s41021-020-00161-3

**Published:** 2020-06-10

**Authors:** Ee Ling Siew, Ahmad Faizzudin Farris, Noramiwati Rashid, Kok Meng Chan, Nor Fadilah Rajab

**Affiliations:** 1 Biocompatibility and Toxicology Laboratory, Centre for Research and Instrumentation Management (CRIM), Universiti Kebangsaan Malaysia, Bangi, Malaysia; 2 Biomedical Science Programme, Faculty of Health Sciences, Universiti Kebangsaan Malaysia, Kuala Lumpur, Malaysia; 3 Environmental Health & Industry Safety Programme, Faculty of Health Sciences, Universiti Kebangsaan Malaysia, Kuala Lumpur, Malaysia; 4 Center for Toxicology and Health Risk Studies, Faculty of Health Sciences, Universiti Kebangsaan Malaysia, Kuala Lumpur, Malaysia; 5 Center for Healthy Aging and Wellness Faculty of Health Sciences, Universiti Kebangsaan Malaysia, Kuala Lumpur, Malaysia

**Keywords:** Clastogenicity, Cytotoxicity, DNA damage, Gadolinium (III) chloride

## Abstract

**Background:**

Rare earth minerals of the lanthanide series are widely used in the field of medical and clinical application. Gadolinium (Gd), the most preferred rare earth mineral is frequently used as magnets, superconductors and magnetic resonance imaging (MRI) contrast agent. Increasing production of gadolinium waste, known potent toxicity of this element and lack of information on its Material Safety Data Sheet (MSDS) prompts health risk assessment on gadolinium. In this study, cytotoxicity and genotoxicity of Gadolinium (III) chloride (GdCl_3_) were investigated using MTT assay, Alkaline Comet assay and Micronucleus assay, respectively.

**Results:**

Our results demonstrated that the viability of GdCl_3_ treated V79–4 cells was significantly (*p* < 0.05) reduced at 1.0 mM after 24 h of incubation. However, no IC50 values were obtained. GdCl_3_ showed no significant (*p* > 0.05) DNA damage both in the presence and absence of metabolic activation. However, it induced significant (*p* < 0.05) clastogenic effect in V79–4 cells at 1.0 mM in the absence of metabolic activation. The clastogenic effect was also seen in the presence of metabolic activation at 0.25 mM, 0.5 mM and 1.0 mM.

**Conclusion:**

Taken together, our study indicated that GdCl_3_ had no cytotoxic effect and does not induce DNA damage. However, this study supports that GdCl_3_ is a probable clastogen. Further studies are needed to investigate the effect of free gadolinium ion (Gd^3+^) for risk assessment on human health.

## Introduction

In this modern age of globalization and technology, the demand for rare earth minerals has significantly increased throughout the years in developing countries such as Malaysia. This is particularly driven by the “green economy” agenda which pushes countries such as Malaysia towards an environment friendly economy [1].

Rare earth minerals are elements of group 15 lanthanide series such as scandium and yttrium. These elements are called rare earth because they are hard to find as a single element. Gadolinium is also one of the elements found in the group 15 lanthanide series. It has an atomic number of 64 and has paramagnetic properties [2]. Due to its properties, gadolinium has been widely used in the industry of superconductors and magnet production. It is also widely used in biomedical field for magnetic resonance imaging (MRI) contrast in combination with chelating fluoride element such as chloride. Chloride use reduces its toxicity and forms gadolinium (III) chloride. Upon metabolism, gadolinium (III) chloride (GdCl_3_) forms free gadolinium ion (Gd^3+^) and three free chloride ions (Cl^−^). Studies conducted by Port et al. in 2008 suggested that free gadolinium ions may exert toxicity risks towards the heart, lungs and brain [3]. Such toxicity might occur because Gd^3+^ has the same ionic radius as calcium ion where it can act as an inorganic blocker to several voltage-channel gates ranging from nano- to micromolar [3]. Gadolinium also showed tendency to inhibit sarcoplasmic reticulum activities in skeletal muscles and reduce the reticuloendothelial system functions in this same study [3]. Yonxing et al. [4] have previously reported that gadolinium also has the tendency to cause DNA breakage and may act as a possible mutagen.

In Malaysia, a rare earth mineral refinery called Lynas collects minerals from Mount Weld, Australia and processes them at Kilang Lynas, in Gebeng, Kuantan to obtain valuable rare earth metals. However, the waste produced from this industry, may cause probable risk towards human health and thus did not obtain the support from the surrounding population [5].

Based on studies regarding the toxicity of free gadolinium ion, the risk towards human health needs to be assessed. Material Safety Data Sheet (MSDS) did not provide any data or even comprehensive report on GdCl_3_. Owing to the absence of such data, this study investigated the clastogenicity and DNA damage abilities of this GdCl_3_ in vitro.

## Materials and methods

### Cell culture

Chinese hamster lung V79–4 fibroblast cells were obtained from ATCC (Rockville, MD) and cultured in Dulbecco’s modified Eagles’s medium (DMEM) supplemented with 10% foetal bovine serum (FBS). Cells were maintained at 37 °C in a 5% CO_2_ incubator. Cells were detached via trypsinization (0.025% trypsin) after reaching confluency, and were ready to be used for tests.

### Preparation of compound

Gadolinium (III) chloride (GdCl_3_) used in this study was purchased from Sigma Aldrich, United States. The dimethyl sulfoxide (DMSO) was used to dissolve this compound as it does not support growth of microbes. The compound was dissolved in DMSO at 250 mM concentration as stock solution and stored at − 20 °C.

### MTT viability assay

MTT (3-[4,5-dimethylthiazol-2-yl]-2,5 diphenyl tetrazolium bromide) assay was used to measure the cytotoxicity of gadolinium (III) chloride by measuring the amount of formazan crystals formed through a reduction process in the mitochondria [6]. Briefly, cells were seeded in 96-well microplate at 5 × 10^4^ cells/mL and were incubated at 37 °C in 5% CO_2_ for 24 H*. media* was replaced treatment medium containing highest concentration of GdCl_3_ (1 mM) on the following day and the cells were incubated further for 24 h. Thirty microliter of sterile MTT solution (5 mg/mL) was added into each well and the plate was incubated for 4 h*. media* and MTT solution were removed after 4 h and 200 μL of DMSO was added into each well to dissolve the formazan crystals. The plate was shaken for 10 min and the optical density reading of each well was obtained using ELISA plate reader at 570 nm wavelength. Graph of viability was plotted against concentration.

### Preparation of S9 mixture

10% (v /v) S9 mixture (the S9 fraction (MOLTOX™, Inc., North Carolina) was used at 1%, final concentration in medium) was prepared fresh during each experiment. The cofactor solution was prepared by dissolving 0.02 g magnesium chloride (MgCl_2_) (8 mM), 0.06 g potassium chloride (KCl) (33 mM), 0.13 mL glucose-6-phosphate (G-6-P) and 1 mL nicotinamide adenine dinucleotide phosphate (NADP) into 25 mL 15 mM phosphate buffer. The stock of cofactor solution was stored at − 80 °C. Subsequently, the S9 was added to the thawed cofactor solution in a ratio of 1: 9. The S9 mixture was filtered using 0.2 μm membrane filter paper and the mixture was kept at 4 °C prior to use.

### Alkaline comet assay

Alkaline Comet Assay was used to assess strand breaks in DNA indicative of DNA damage [7]. Cells seeded in 6-well plate were treated with 0.25 mM, 0.5 mM and 1.0 mM GdCl_3_ for either 3 h or 24 h of incubation with or without metabolic activation. Cells were also treated with hydrogen peroxide (H_2_O_2_) (positive control, 1.0 mM) for 30 min. Following respective incubations, detached cells in the media were collected, added to trypsinized cells and centrifuged (2500 rpm/5 min). The supernatant was removed and the pellet was washed with Ca^2+^−/Mg^2+^-free PBS prior to re-centrifugation. Pellets left at the bottom were mixed thoroughly with 80 mL of 0.6% low melting agarose (LMA) (w/v) and the mixture was pipetted onto hardened 0.6% normal melting agarose (NMA) (w/v) on the slide. Cover slips were placed to spread the mixture and the slides were left on ice for LMA to solidify. Following the removal of the cover slips, the embedded cells were lysed in lysis buffer containing 2.5 M NaCl, 100 mM Na_2_EDTA, 10 mM Tris, and 1% Triton X-100 for 1 h at 4 °C. Slides were soaked in electrophoresis buffer solution for 20 min at 4 °C for DNA-unwinding before electrophoresis at 300 mA, 25 V, for 20 min. Subsequently, the slides were rinsed with neutralizing buffer for 5 min and stained with 50 mL ethidium bromide solution. Slides were left overnight at 4 °C before analysis with Leitz Laborlux epifluorescence microscope equipped with 515 barrier filter and 560 emission filter. Fifty cells per slide were scored and the tail moment (Tm) and % tail DNA (TD) were analyzed with COMET assay III (Perceptive Instruments, UK).

### Micronucleus assay

Clastogenicity was assessed using the previously described micronucleus assay [8]. Briefly, 2 mL of cell suspension (1 × 10^5^ cells/mL) were seeded in 6-well plates. The plates were incubated for 24 h at 37 °C supplemented with 5% CO_2_. After 24 h, the media was replaced with 2 mL of treatment medium containing three suitable concentrations obtained from the MTT assay. In the absence of metabolic activation, treatment was given for 3 followed by 21- h- recovery period or 24 h whilst it was only given for 3 h in the presence of metabolic activation. After each treatment, the cells were harvested and cell count was conducted to obtain values for Relative Increase in Cell Count (RICC) and Relative Population Doubling (RPD). After harvest, the cell pellet obtained was added with 2 mL of 75 mM KCl and left for 5 min. Fixation was then conducted by adding 5 mL of Clarke’s Fixative Solution (3-parts methanol to 1-part acetic acid) and the solution was immediately centrifuged at 1500 rpm for 5 min. After centrifugation, the supernatant was removed and replaced with 5 mL of fixative solution and was left for another 30 min before centrifugation. The process was repeated again however the solution was left for only 10 min before centrifugation. After the final centrifugation, the supernatant was removed, leaving approximately 2 mL of solution. Around 2–3 drops of the cell solution was dropped onto end-frosted microscope slides pre-warmed at 35 °C and were left to dry. The slides were stained with few drops of acridine orange (0.02 mg/mL) and was observed under fluorescent microscope with emission rate of 450–490 nm. Frequency of micronucleus formed was plotted in a graph against concentration or controls.

### Statistical analysis

The data were presented as the mean ± standard error of mean (SEM) at least three independent experiment. Statistical significance between means was assessed using ANOVA followed by a Dunnet’s t-test. A *p*-value of < 0.05 was considered significant.

## Results

### MTT viability assay

**Fig. 1:**
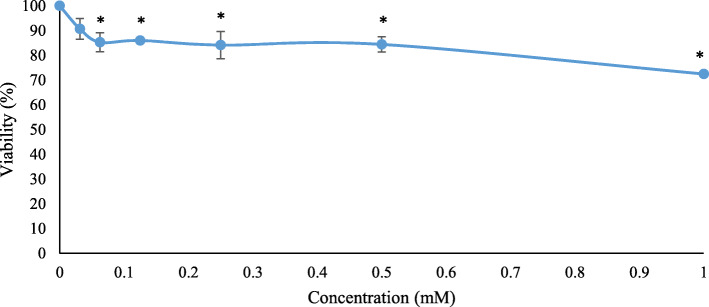
Cytotoxicity of GdCl_3_ against V79–4 cells with 24 h of incubation. The results are expressed as mean ± SE of at least three independent experiment. *Significant difference (*p* < 0.05) as compared to negative control

Figure 1 shows the cytotoxic effects of GdCl_3_ assessed using MTT assay after 24 h of incubation. V79–4 cell viability showed a significant decrease (*p* < 0.05) from 0.06 mM to 1.0 mM of GdCl_3_.

### Alkaline comet assay

Alkaline Comet assay was employed to detect DNA damage induced by GdCl_3_ in V79–4 cells. Low level of background damage was seen in the negative control cells. Scoring for the DNA damage was done based on the tail moment (Tm) and % tail DNA (TD) values.

As shown in Table 1, there was slight increase with no significant difference in Tm and TD at all three concentrations, (0.65 ± 0.16) and (4.69 ± 1.08), (0.44 ± 0.19) and (4.27 ± 1.24), and (1.05 ± 0.14) and (7.04 ± 0.90) of GdCl_3_ treated cells respectively at 3 h without S9 metabolic activation as compared to negative control cells. Similar profile was also seen following treatment for 24 h. Furthermore, there was no further increase in DNA damage as shown by Tm and TD on GdCl_3_ treated cells at 3 h and 24 h with S9 metabolic activation, as shown in Table 2. These results indicate that GdCl_3_ has no capability to induce direct DNA strand breakage in V79–4 cells. H_2_O_2_ treatment at 1 mM s used as positive control caused significant (*p* < 0.05) increase in DNA damage shown by Tm and TD (Table 1 & Table 2).

**Table 1:** Level of DNA damage on V79–4 treated cell line without metabolic activation. The results are the means ±SE of at least three separate experiments (*p < 0.05 versus negative control)

Level of DNA damage (Arbituary unit)		
	***Tm***	***TD***
	***3 h***	
Negative control	0.31 ± 0.08	2.90 ± 0.63
0.25 mM GdCl_3_	0.65 ± 0.16	4.69 ± 1.08
0.5 mM GdCl_3_	0.44 ± 0.19	4.27 ± 1.24
1.0 mM GdCl_3_	1.05 ± 0.14	7.04 ± 0.90
	***Tm***	***TD***
	***30 min***	
Positive control	*7.92 ± 0.49	*32.42 ± 2.34
	***Tm***	***TD***
	***24 h***	
Negative control	0.60 ± 0.07	5.11 ± 0.57
0.25 mM GdCl_3_	0.77 ± 0.20	6.49 ± 1.62
0.5 mM GdCl_3_	0.39 ± 0.03	3.75 ± 0.12
1.0 mM GdCl_3_	1.24 ± 0.31	8.85 ± 1.93
	***Tm***	***TD***
	***30 min***	
Positive control	*6.00 ± 0.81	*29.49 ± 3.38

**Table 2:** Level of DNA damage on V79–4 treated cell line with metabolic activation. The results are the means ±SE of at least three separate experiments (*p < 0.05 versus negative control)

Level of DNA damage (Arbituary unit)		
	***Tm***	***TD***
	***3 h***	
Negative control	0.42 ± 0.12	3.46 ± 0.72
Negative control + S9	0.47 ± 0.09	3.80 ± 0.66
0.25 mM GdCl_3_	0.61 ± 0.06	4.70 ± 0.30
0.5 mM GdCl_3_	0.98 ± 0.07	6.93 ± 0.34
1.0 mM GdCl_3_	0.54 ± 0.08	3.81 ± 0.63
	***Tm***	***TD***
	***30 min***	
Positive control	*6.87 ± 0.88	*26.40 ± 2.14
	***Tm***	***TD***
	***24 h***	
Negative control	0.42 ± 0.12	3.44 ± 0.45
Negative control + S9	0.66 ± 0.02	5.31 ± 0.50
0.25 mM GdCl_3_	1.15 ± 0.2	7.25 ± 1.25
0.5 mM GdCl_3_	0.97 ± 0.24	6.83 ± 1.46
1.0 mM GdCl_3_	1.32 ± 0.04	7.48 ± 0.47
	***Tm***	***TD***
	***30 min***	
Positive control	*6.87 ± 0.88	*26.40 ± 2.14

### Micronucleus assay

**Fig. 2:**
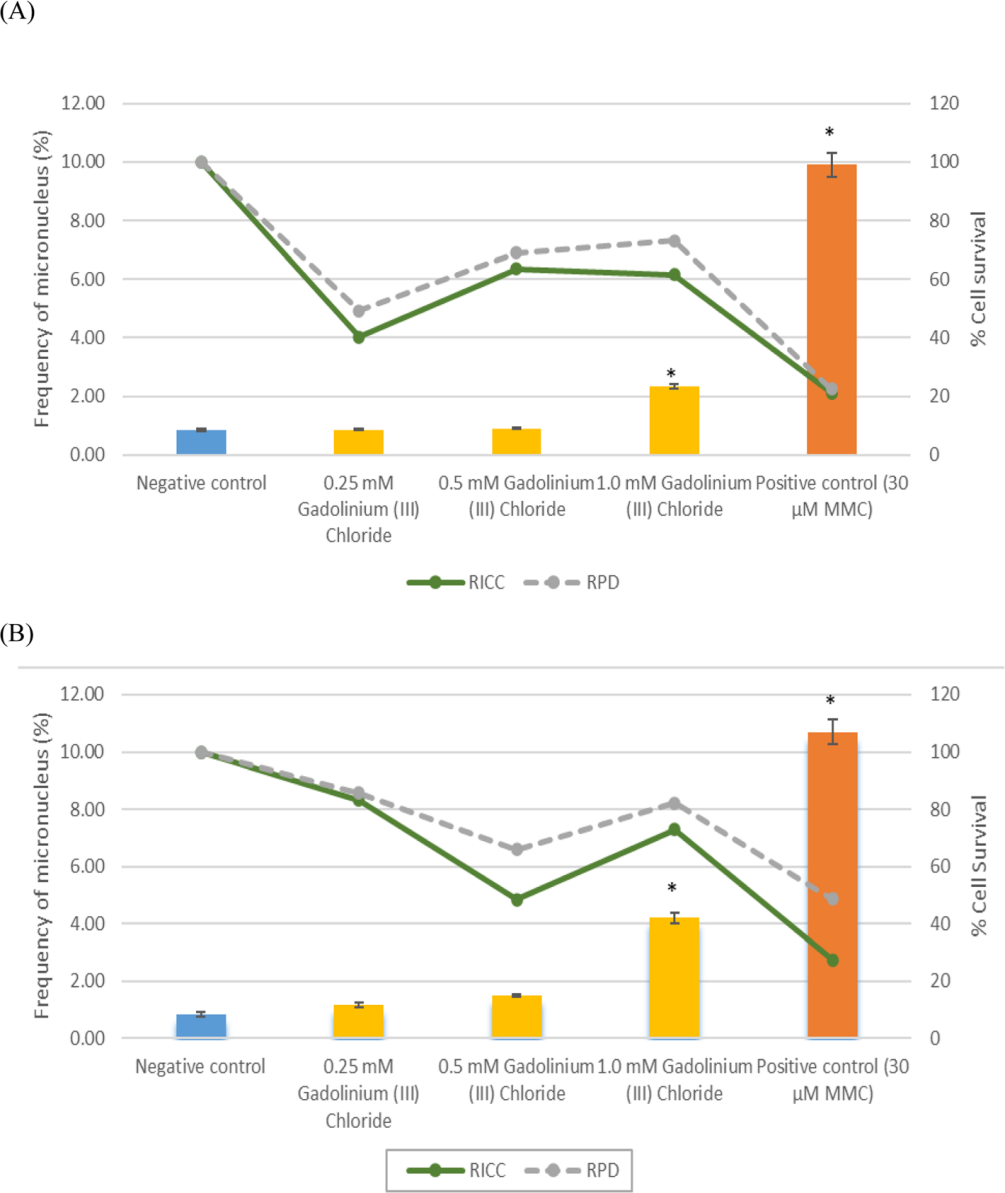
Percentage of micronucleus, RICC and RPD, in V79–4 cells induced by GdCl_3_ and positive control (Mitomycin C) following (**a**) 3 h, **b** 24 h without metabolic activation. The results are the means ±SE of at least three separate experiments (*p < 0.05 versus negative control)

Formation of micronucleus in V79 fibroblast cells induced by GdCl_3_ was investigated using acridine orange dye for observation. Micronuclei in V79–4 cells were scored under the fluorescence microscope. Our data showed a relative increase in cell count (RICC) and relative population doubling (RPD) for V79–4 cells following treatment for 3 h and 24 h without S9 metabolic activation, as shown in Fig. 2. Percentage of RICC and RPD both showed an increase across all three concentrations of GdCl_3_.

**Fig. 3:**
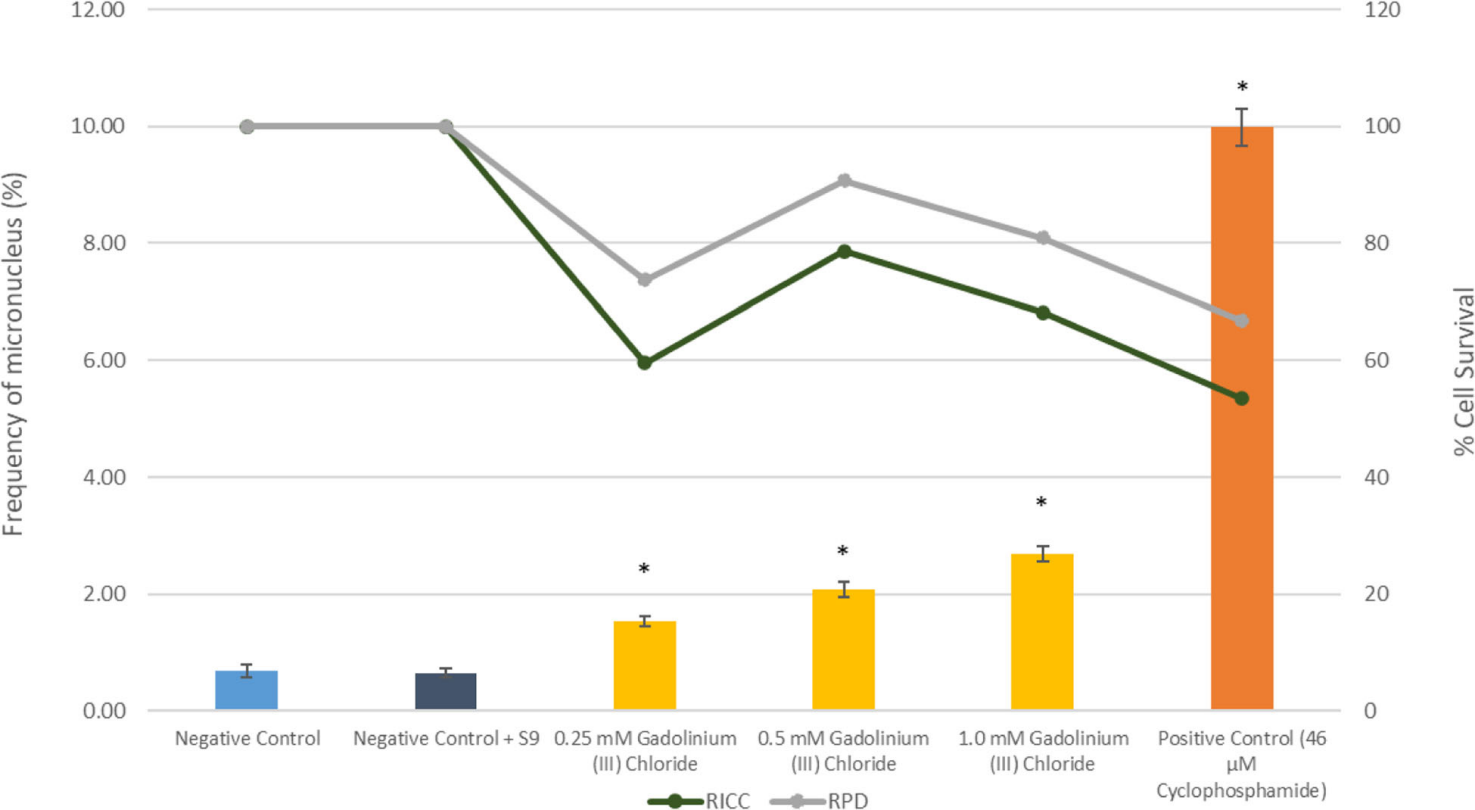
Percentage of micronucleus, RICC and RPD, in V79–4 cells induced by GdCl_3_ and positive control (Cyclophosphamide) following 3 h with metabolic activation. The results are the means ±SE of at least three separate experiments (*p < 0.05 versus negative control)

Figure 3 shows the relative increase in cell count (RICC) and relative population doubling (RPD) for V79–4 cells after 3 h of treatment following S9 metabolic activation. There was a slight increase in percentage frequency of micronucleus formation in V79–4 cells after treatment for 3 h and 24 h without metabolic activation for all three concentrations respectively as shown in Fig. 2. However, only 1.0 mM GdCl_3_ showed significant difference (*p* < 0.05) as compared to the negative control.

Following S9 metabolic activation, all three concentrations showed significant difference in percentage frequency of micronucleus formation (p < 0.05) as compared to negative control (Fig. 3). This result indicates that GdCl_3_ treated V79 cells has clastogenic potential with direct capability to induce chromosomal damage.

## Discussion

This study investigated the cytotoxicity and genotoxicity of GdCl_3_ in V79–4 cells. Usage of GdCl_3_ in biomedical applications such as MRI contrasts increases the need for chemical hazard risk assessment. Even though normal physiological excretion in humans may eliminate GdCl_3_ wastes to a certain degree, GdCl_3_ wastes may still accumulate in various organs and tissues. Upon accumulation, GdCl_3_ is metabolically activated to form free gadolinium ions which induce significant toxicity in humans [9].

MTT assay was used as analysis of cytotoxicity assay, this study used 1.0 mM GdCl_3_ as the highest concentration based on previous study conducted by Mowat et al. [10]. They reported that the concentration of gadolinium in the cells is directly proportional to the concentration of gadolinium in the incubation media; whilst using 0.5 mM till 2.0 mM as the optimum range for GdCl_3_ [10]. Consistent with this, 1.0 mM GdCl_3_ was chosen because it is considered the median concentration based on the reported range. Our data demonstrated that no IC50 values were obtained after treatment with GdCl_3_. This is consistent with reports from Liu et al. [11] whereby gadolinium did not cause cell death and had good biocompatibility towards cells. According to De Siervi et al. [12], any compound which shows a viability up to 30% or more as compared to the original cell value indicate cytotoxic effect. It is possible that gadolinium did not affect mitochondria but may affect other organelles that cause cell death. Three analyzable concentrations (0.25 mM, 0.5 mM and 1.0 mM) were chosen from the MTT assay and were used for subsequent analyses.

Alkaline Comet assay was used to investigate the single strand breaks in DNA [7]. Tm and TD values showed no significant increases in DNA damage among cells treated with GdCl_3_ as compared to negative control both in presence and absence of metabolic activation. This data suggested that gadolinium do not directly affect the DNA and cause its damage.

Chromosome aberrations were also analyzed in metaphase stage as part of a cytogenetic study [13]. RICC and RPD values recorded in our results were used to avoid false negative data by confirming that the cell has undergone division (OECD 487, 2016). Indeed, RICC and RPD values did not exceed those values obtained from negative control confirming that the data obtained was valid. This adheres to guidelines set by OECD 487 [8] which states that a value for RICC and RPD must not exceed the negative control to confirm the validity of the data.

In this study, our results showed a significant increase in micronuclei formation at 1.0 mM GdCl_3_ after 3 h and 24 h of incubation without S9 metabolic activation. Clearly, this indicates that GdCl_3_ induces clastogenicity in these cells. Although GdCl_3_ requires metabolic activation, our data showed that GdCl_3_ increases micronuclei formation at as low as 0.25 mM concentration without metabolic activation. Thus, this results showed that free gadolinium ion may directly cause clastogenicity in the cells. This scenario might be due the fact that GdCl_3_ acts as a DNA intercalator specifically in the DNA bases and its trigonal planar structure. In another study also reported that any chemical compound with a planar topology may act as DNA intercalator in the DNA bases [14]. In addition, gadolinium may also act the same as histone acetylation. When a histone is acetylated, the absorption ability of the chromatin increases and causes nucleosome loosening [15]. This strongly supports that such process may enable the process of chromatin repair mechanism and increase the risk of mutagenesis.

## Conclusions

This study provides evidence that GdCl_3_ do not induce cytotoxic effect and DNA damage in V79–4 cells. However, it has potential to cause clastogenicity and may contribute to adverse health effects. Further investigations are needed to specifically elucidate and assess risk of free gadolinium ion (Gd^3+^) towards human health.

## Data Availability

Not applicable.
